# Advances in artificial vision systems: a comprehensive review of technologies, applications, and future directions

**DOI:** 10.1007/s13534-025-00513-4

**Published:** 2025-10-23

**Authors:** Jisung Kim, Jong-Mo Seo

**Affiliations:** 1 Biomedical Research Institute, Seoul National University Hospital, Seoul, South Korea; 2 Department of Electrical and Computer Engineering, Seoul National University, Seoul, South Korea

**Keywords:** Artificial vision, Retinal prosthesis, Nerve stimulation, Implantable medical device

## Abstract

This review article focuses on recent advancements and persistent challenges in artificial vision prostheses designed to restore sight for patients affected by retinal diseases. It comprehensively examines various approaches, including epiretinal, subretinal, and suprachoroidal implants, as well as optic nerve and visual cortex stimulation strategies. The critical role of the retina in visual perception is explored, emphasizing how retinal degeneration affects the transmission of visual information and how artificial devices aim to replicate this function. The review also discusses the technological complexities of artificial retina development, particularly challenges associated with enhancing resolution, minimizing the spread of electrical stimulation, and achieving reliable long-term device functionality within the biological environment. Practical clinical outcomes, such as surgical feasibility, device durability, and biocompatibility, are analyzed in light of these innovations. Furthermore, emerging trends are highlighted, including the adoption of flexible materials, photovoltaic structures, and 3D electrode architectures to improve the performance and longevity of implants. Ultimately, future advancements in artificial vision systems will depend on integrated approaches that combine cutting-edge engineering with a deep understanding of biological systems to achieve meaningful and lasting visual restoration.

## Introduction

In recent years, rapid advancements in electronics have driven the development of artificial visual transmission devices aimed at restoring vision for the blind. These devices utilize bionics, a fusion of biology and electronics, with the primary goal of replicating retinal function.

The retina is a transparent nervous tissue that occupies approximately two-thirds of the interior of the eyeball and is located in the posterior segment [1–4]. Pigment cells and choroid underneath the retina support its physiological milieu. The retina is instrumental in visual perception. Light entering the eye through the cornea and lens forms an image on the retina that is then converted into neural signals by photoreceptors. Bipolar, amacrine, horizontal, and ganglion cells make up the neural network for intraretinal signal processing, such as color, edge, and motion recognition. The processed vision information is then delivered to the visual cortex via the lateral geniculate nucleus. The optic nerve consists of axons of the retinal ganglion cells, unmyelinated on the retinal surface and myelinated when they pass through the lamina cribrosa of the optic nerve head. Color-sensing cone cells are concentrated in the fovea, which is characterized by high resolution and sensitivity. The macula, which surrounds the fovea, is central to the field of vision, and the peripheral retina enables the perception of dimly lit and peripheral visual fields [5–9].

If the photoreceptors cannot convert light to neural signals, the patient cannot see even though there are remaining retinal neural cells. Retinitis pigmentosa (RP) and age-related macular degeneration (AMD) are well-known conditions of photoreceptor degeneration or damage. AMD associated with abnormal new vessel growth under the photoreceptor layer can be alleviated by intraocular injection of vascular endothelial growth factor inhibitors nowadays, but there is still no appropriate treatment for RP. The artificial visual transmission device may be the solution to this unwanted condition [10–13].

## Artificial vision prosthesis

### Stimulation sites for artificial vision

**Fig. 1:**
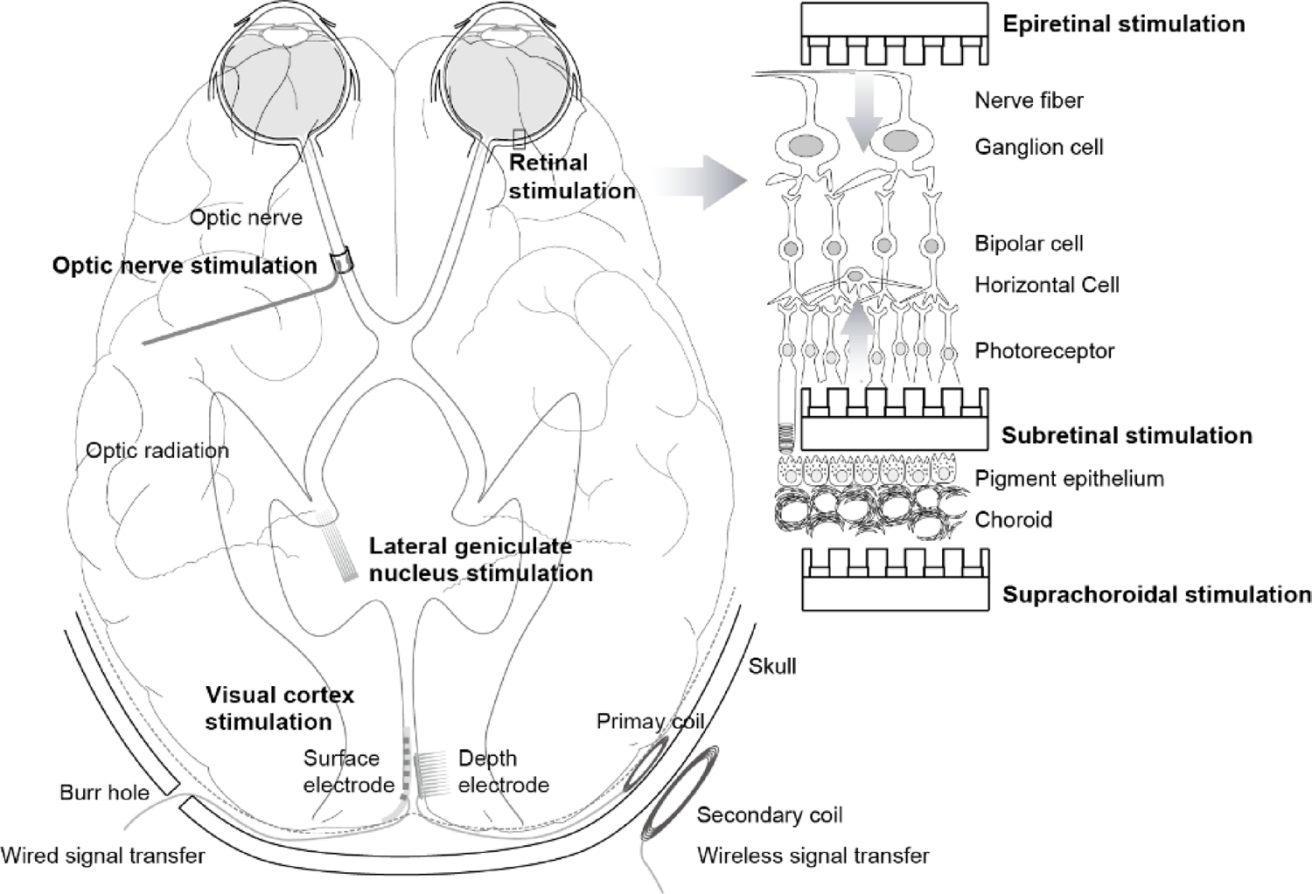
Processing of visual information and stimulation sites in the visual pathway, including a cross-sectional structure of the retina showing electrode implantation targets (Reprinted from Seo et al. [13])

Four sites on the visual pathway are considered as targets for vision prostheses: the retina, optic nerve, lateral geniculate nucleus, and visual cortex (Fig. 1). The optic nerve seems to be a good candidate when viewing the simplified visual pathway diagram, but the optic nerve bundle, comprising approximately 1.2 million fibers, presents a challenge due to its limited diameter of 4–5 mm, which restricts the achievement of high spatial resolution. Additionally, approaching the bony optic canal requires a neurosurgical approach. The lateral geniculate nucleus and visual cortex necessitate craniotomy for electrode implantation, posing accessibility challenges due to their deep brain location and the intricate surface of the visual cortex within the occipital lobe. The retina has remaining neurons such as bipolar and ganglion cells even after photoreceptor degeneration, and these cells can be the target for stimulation. According to the implantation site of the stimulation device, epiretinal (over the retina), subretinal (under the retina), and suprachoroidal (between the choroid and sclera) approaches are under investigation [14–19].

### Epiretinal prosthesis

Epiretinal placement of the stimulation device seems to be easy, requiring only vitrectomy and attaching the device over the retina [18, 20–22]. However, simple attachment cannot ensure even and close placement of the device over the retina, and an increase in the distance between the device and the remaining retinal neurons will increase the impedance of the stimulation electrode. This induces an increase in the stimulation current and the risk of heat accumulation inside the eye. For instance, in the case of the Argus II electrode, which employs flexible polyimide, it has been observed that the gap between the electrode and the retina tends to widen post-implantation. Addressing these challenges requires the development of new materials that maintain consistent contact and adhere to the curved inner wall of the eyeball, thereby mitigating the risks of device displacement and uneven stimulation [23].

#### Argus II (Second Sight, USA)

Second Sight Medical Products, founded in the United States, developed the Argus II system, which became the first FDA-approved retinal prosthesis in 2013 and achieved CE marking for commercialization in Europe. Following its approval, Argus II was implanted in over 350 patients worldwide, marking a major milestone in the clinical application of visual prosthetics.

The Argus II system consists of both external and internal components. Externally, a miniature video camera mounted on a pair of glasses captures visual information, which is processed by a portable video processing unit. The processed signals are wirelessly transmitted to the internal implant, where a 60-electrode flexible polyimide array is placed epiretinally over the macula. The electrodes electrically stimulate the underlying retinal ganglion cells, generating visual percepts perceived by the user.

Clinical trials in [24–26] demonstrated that Argus II provided users with the ability to perceive light, detect motion, and recognize large objects. Although the system enabled functional improvements in daily activities, the maximum visual acuity achieved was limited to around 20/1262, remaining below the threshold for legal blindness.

Furthermore, the relatively low electrode density constrained the spatial resolution, limiting the ability to distinguish fine details.

**Fig. 2:**
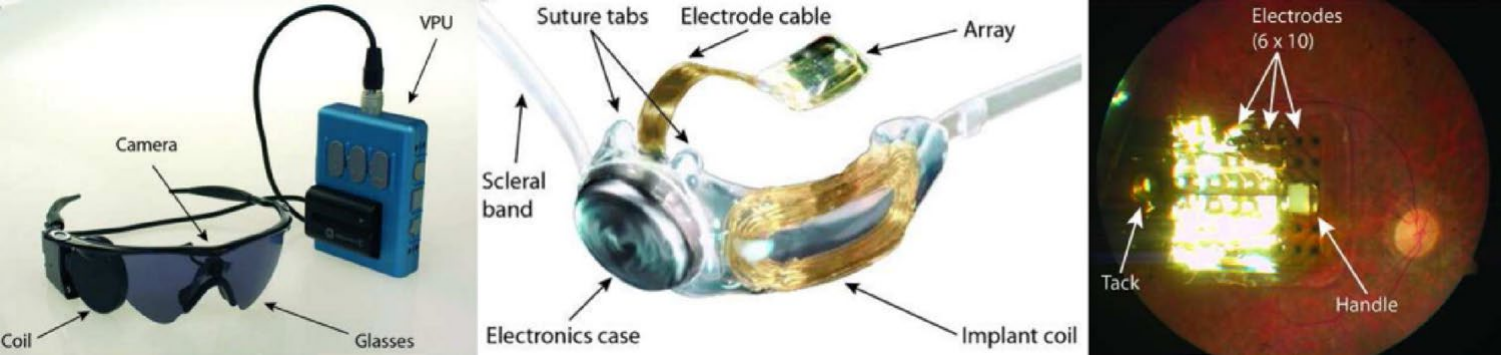
The Argus II retinal prosthesis system, showing **A** the external components with glasses-mounted camera, external coil, and video processing unit; **B** the internal implant with receiving coil and electrode array; and **C** the epiretinal electrode array fixed to the macula with a tack (Reprinted from Humayun et al. [27], with permission from Elsevier)

Several challenges also emerged during long-term follow-up, including mechanical failures, conjunctival erosion, and retinal damage associated with the surgical implantation and chronic presence of the device. Additionally, the requirement for bulky external hardware and alignment between the camera and the user’s intended gaze posed ergonomic and functional limitations (Fig. 2).

Despite these limitations, as reported in [27–29], Argus II represented a landmark achievement by demonstrating the feasibility of partial vision restoration in patients with profound vision loss. Its development laid the foundation for future retinal prostheses and provided invaluable clinical data regarding patient adaptation, surgical outcomes, and device longevity.

#### Iris II (Pixium Vision, France)

Pixium Vision, a French company specializing in retinal prosthetics, developed the IRIS® II system as an advancement of previous epiretinal devices. Designed to address limitations observed in earlier systems, IRIS II incorporated 150 electrodes, achieving approximately 2.5 times the resolution of the Argus II system.

**Fig. 3:**
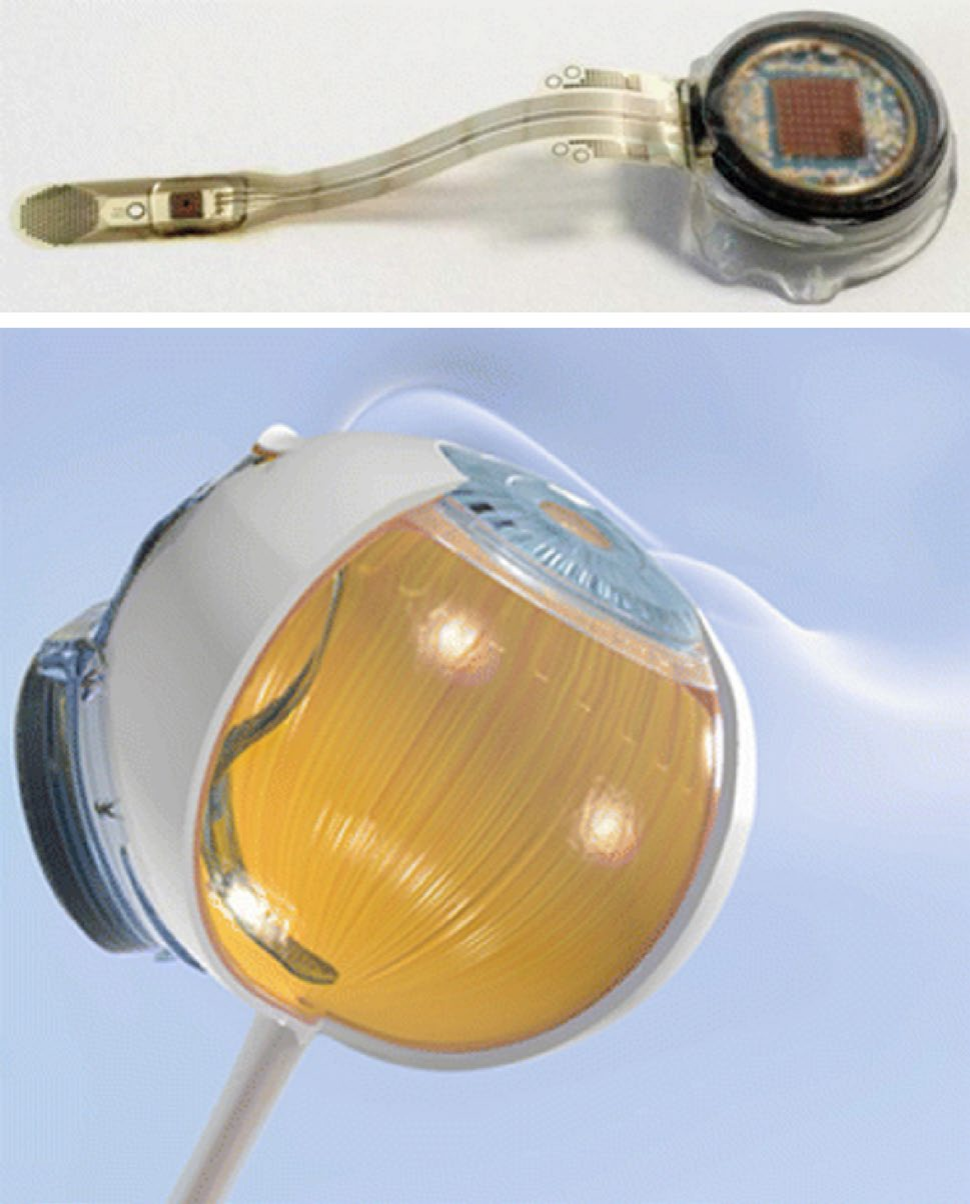
The IRIS II retinal prosthesis system, showing **A** the implant with circular ASIC and polymer-based electrode array and **B** the external and internal system components (Reprinted from Hornig et al. [30], with permission from Springer Nature)

As shown in Fig. 3, the IRIS II system consists of a bio-inspired external camera integrated into glasses that dynamically captures the visual scene. The captured images are processed by a portable visual processing unit and then wirelessly transmitted to the retinal implant. The epiretinal implant, composed of 150 stimulating electrodes arranged on a flexible polymer substrate, directly stimulates surviving retinal ganglion cells.

The modular design of IRIS II facilitated easier upgrades and replacements of system components, promoting flexibility and potential longevity of the device. Clinically, patients implanted with IRIS II in 2017 [30, 31], demonstrated improvements in basic light perception and the ability to detect moving objects. However, visual acuity remained limited, and challenges similar to those faced by the Argus II system persisted, such as variability in electrode-retina contact and limited fine detail recognition.

Despite these challenges, the IRIS II system contributed valuable insights into the optimization of electrode array design, modular prosthesis systems, and patient rehabilitation strategies. Furthermore, knowledge gained from the development of IRIS II informed Pixium Vision’s subsequent efforts toward more advanced subretinal prosthesis designs.

#### NR600 (Nano Retina, Israel)

Nano Retina, an Israeli company specializing in neurostimulation technologies, developed the NR600 system as an innovative epiretinal prosthesis featuring three-dimensional (3D) needle-like electrodes. Unlike traditional flat electrode arrays, the NR600 utilizes 3D penetrating electrodes designed to insert into the retinal surface, providing closer proximity to target neurons and reducing the stimulation threshold.

The NR600 system comprises a dense electrode array with 676 individual microelectrodes, each measuring approximately 150 ± 30 µm in length, with an exposed tip height of around 50 µm. This configuration enables more localized stimulation of inner retinal neurons while minimizing current spread. Power and data are wirelessly transmitted via a custom-designed pair of rechargeable glasses equipped with an infrared laser link.

Clinical evaluations of the NR600 indicated improved stimulation efficiency compared to conventional epiretinal devices, with patients demonstrating enhanced light perception and spatial localization. Additionally, the system’s wireless architecture eliminated the need for trans-scleral wires, reducing surgical complexity and postoperative complications.

However, mild side effects such as transient corneal edema, moderate increases in intraocular pressure, and occasional intraocular lens displacement have been observed during clinical studies. These findings underscore the importance of ongoing refinement in surgical techniques and implant biocompatibility to optimize clinical outcomes in 2022 as shown in [32].

The NR600’s novel approach to epiretinal stimulation, through 3D penetrating electrode arrays, offers a promising avenue for achieving higher spatial resolution and more naturalistic visual restoration in future retinal prostheses.

#### iBIONICS (iBIONICS, Canada)

iBIONICS, a Canadian company originating from research conducted at the University of Melbourne, developed a novel retinal prosthesis system leveraging the unique properties of diamond materials in 2020. The device features a 256-electrode array fabricated from diamond, where each electrode measures approximately 120 µm × 120 µm with a 30 µm spacing between adjacent electrodes.

Diamond offers exceptional biocompatibility, chemical stability, and electrical conductivity, making it an ideal material for chronic implantation within the ocular environment. The high mechanical strength of diamond ensures durability, while its bio-inert nature minimizes inflammatory responses, promoting long-term device stability.

According to [33, 34], the iBIONICS system aims to deliver higher resolution stimulation compared to previous generations of epiretinal prostheses. Its densely packed electrode array enables more localized activation of retinal ganglion cells, theoretically improving spatial resolution and contrast sensitivity. Early preclinical results demonstrated effective charge injection and stable performance without significant adverse tissue reactions.

Although clinical trials have not yet been widely reported, the iBIONICS approach represents a significant advancement in materials engineering for retinal prostheses. Ongoing development focuses on optimizing electrode–electrolyte interfaces, ensuring long-term operational stability, and enhancing surgical techniques for precise implantation.

By integrating advanced materials like diamond into prosthetic designs, the iBIONICS system pushes the frontier of high-resolution artificial vision, offering the potential for improved outcomes in future clinical applications.

#### IMIE 256 (IntelliMicro Micro Co and Golden Eye Bionic, China and USA)

The Intelligent Micro Implant Eye (IMIE) 256, developed through collaborative efforts to improve epiretinal prosthesis technology, represents a significant advancement in electrode density and implant architecture. The system features a flexible electrode array fabricated on a biocompatible Parylene-C substrate, comprising 248 large electrodes (210 µm diameter) and 8 smaller electrodes (160 µm diameter) strategically arranged to optimize stimulation coverage over the central visual field.

The implant dimensions span approximately 4.75 mm × 6.50 mm, with electrode pitches of 350 µm and 300 µm for the large and small electrodes, respectively. This layout aims to maximize spatial resolution while minimizing overlap of electrical fields. The IMIE 256 array is encapsulated in multilayer corrosion-resistant barriers, promoting mechanical durability and ensuring thermal and electrical safety during chronic implantation.

In 2021 clinical trials of the IMIE 256 [35], reported notable improvements in visual function tests, including light perception, object localization, and basic pattern recognition. However, complications such as minor device displacement toward the superior temporal macula and transient elevations in intraocular pressure following repositioning procedures have been observed, underscoring the importance of long-term follow-up and surgical refinement.

The IMIE 256 exemplifies the ongoing evolution of epiretinal prostheses toward higher resolution, biocompatibility, and long-term stability, providing critical insights for the design of future next-generation visual prosthetic systems.

#### EPI-RET3 (RWTH Aachen University, Germany)

The EPI-RET3 system, developed by researchers at RWTH Aachen University, represents an early generation of wireless epiretinal prostheses designed to simplify implantation procedures and enhance patient comfort. The device consists of a 25-electrode array fabricated on a parylene-C substrate, with each electrode coated in iridium oxide (IrO_x_) and measuring approximately 100 µm in diameter.

EPI-RET3 eliminates trans-scleral wiring by utilizing an inductive wireless link for power and data transmission, reducing the risk of infection and postoperative complications. The device is anchored to the retina using a tack fixation method, which secures the electrode array in place but introduces localized risks such as gliosis and retinal scarring.

Clinical trials with the EPI-RET3 in 2008 [36–38], demonstrated basic light perception and object detection capabilities. However, several complications were reported, including non-progressive epiretinal gliosis at the tack sites, inflammatory responses associated with corneal sutures, minor choroidal atrophy, and retinal tears requiring additional surgical interventions.

Despite its limitations, the EPI-RET3 project contributed valuable insights into wireless implant technologies, electrode-tissue interactions, and the surgical management of retinal prostheses. Lessons learned from EPI-RET3 have influenced the design of subsequent wireless visual prosthetic systems focused on improving safety, durability, and patient outcomes.

### Subretinal prosthesis

Subretinal stimulation represents an alternative strategy to epiretinal approaches by targeting the space between the retinal pigment epithelium (RPE) and the photoreceptor or bipolar cells. This method places electrodes closer to the remaining neural layers, particularly the bipolar cells, which can lead to more naturalistic visual signal processing. The proximity ensures more uniform and lower stimulation thresholds compared to epiretinal systems, where the distance to target neurons often varies post-implantation.

The fundamental advantage of subretinal prostheses lies in their ability to exploit the surviving retinal interneuronal circuitry. After photoreceptor degeneration, as seen in diseases like retinitis pigmentosa, bipolar cells often remain intact and can be stimulated to relay more physiologically processed signals to the brain. This pathway enables the potential for richer and more naturalistic visual perception compared to direct stimulation of ganglion cells.

However, subretinal implantation presents substantial surgical challenges. The process requires delicate detachment of the neurosensory retina from the underlying RPE without inducing irreversible damage. Previously reported [39–43] showed, such detachment can disrupt nutrient and oxygen supply to the outer retina, posing risks of additional degeneration to surviving photoreceptors and RPE cells. Furthermore, maintaining the reattached retina’s integrity over the implanted array demands sophisticated surgical skill and biocompatible materials to prevent chronic inflammation, retinal detachment, or fibrosis.

Despite these hurdles, subretinal prostheses are promising due to their anatomical integration, consistent electrode-tissue distances, and the possibility of restoring partial vision through more natural pathways. Continued advancements in surgical techniques, biocompatible materials, and electrode design are essential for optimizing clinical outcomes and extending the longevity and functionality of these implants as depicted in [44, 45].

#### Alpha IMS (Retina Implant AG, Germany)

Retina Implant AG, a company founded by researchers from the University of Tübingen in Germany, successfully commercialized one of the earliest subretinal prostheses, the Alpha IMS system. Unlike epiretinal prostheses that require external cameras to capture images, Alpha IMS integrated photodiodes and amplification circuits directly onto a flexible polyimide-based microelectrode array, enabling direct light detection through natural eye movements. This design preserved the intuitive coordination between eye and head movements, offering a more physiologically compatible method for visual perception restoration.

**Fig. 4:**
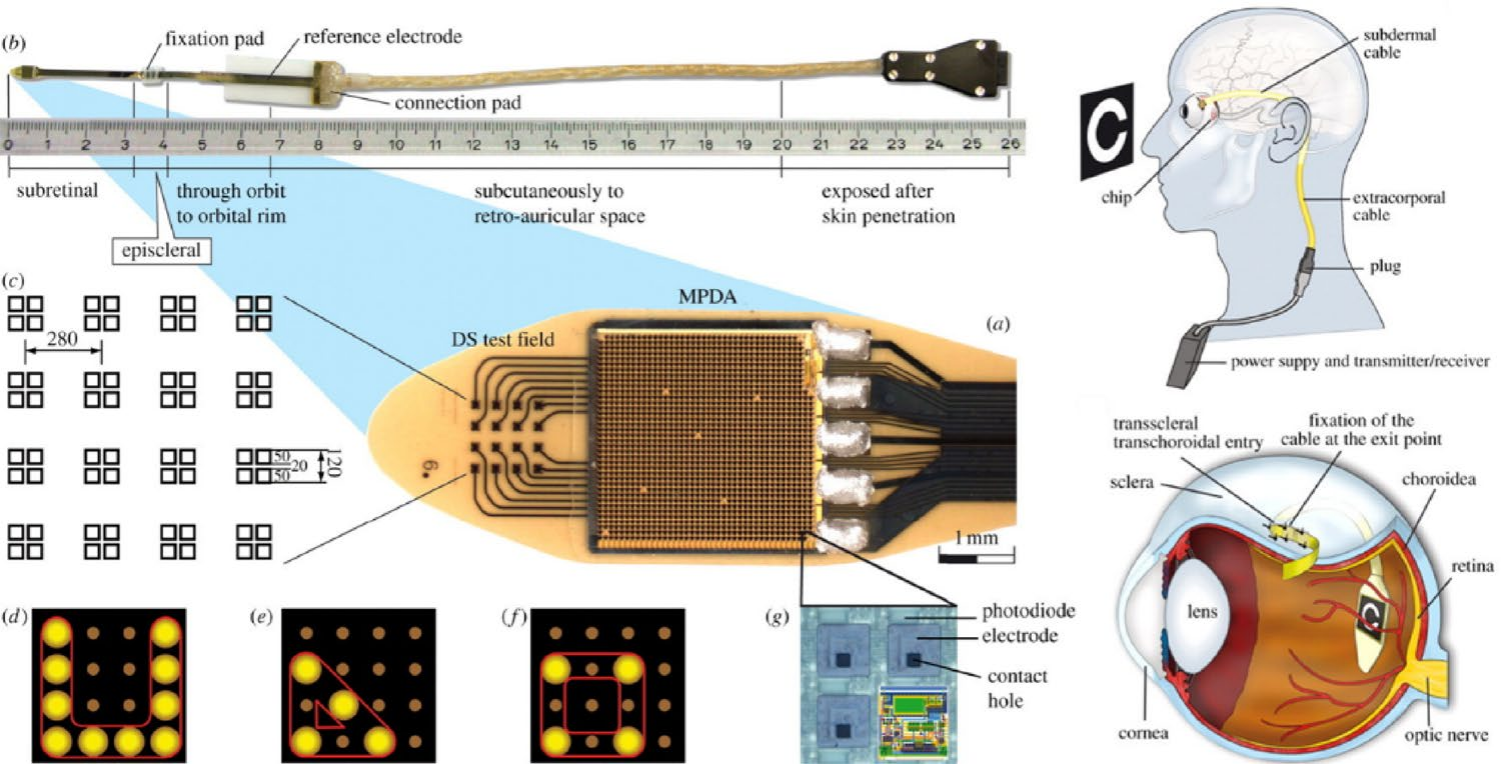
The Alpha IMS subretinal implant consists of a 3.0 × 3.1 mm microphotodiode array (MPDA) with 1500 pixels on a thin polyimide foil, featuring a special field with 16 electrodes for direct stimulation. This foil extends from the eyeball, connecting to a power control unit behind the ear. The implant demonstrates capabilities like pattern formation and shape shifting using electrode stimulation. Detailed are the implant's elements, including photodiodes and amplifier connections. The implant's pathway runs from the eye to a power unit behind the ear, positioned under the retina (Reprinted from Zrenner et al. [46], under the Creative Commons Attribution License)

The Alpha IMS implant features a 3.0 mm × 3.0 mm microchip containing approximately 1500 microphotodiodes, each paired with an amplifier and an electrode. These elements detect incident light and electrically stimulate bipolar cells within the retina. The subretinal location of the device allows it to exploit the surviving inner retinal network, enabling signal processing pathways similar to natural vision. By maintaining proximity to the bipolar cells, the device reduces the required stimulation thresholds and promotes spatially localized activation (Fig. 4).

Clinical trials in [46–48] demonstrated that Alpha IMS in 2011 could restore visual functions such as light perception, object localization, and even letter recognition in some patients. The best-recorded visual acuity achieved was approximately 20/546, roughly twice the performance of the Argus II system. However, this result still fell short of the projected 20/250 acuity based on the electrode size and theoretical pixel density, reflecting the complex biological limitations in translating electrode miniaturization into functional resolution improvements.

While the Alpha IMS system showed promising potential, several challenges remained. Device longevity was limited by the stability of the integrated CMOS circuits within the subretinal environment, and surgical complexity associated with subretinal implantation posed additional barriers to widespread clinical adoption. Moreover, stringent European medical device regulations increased the financial burden on the company. Ultimately, Retina Implant AG ceased operations in 2019. Nevertheless, the Alpha IMS project provided critical insights into subretinal device development as shown in [49–51], including the importance of photodiode stability, energy efficiency, surgical accessibility, and leveraging the intrinsic retinal processing pathways. These contributions continue to inform next-generation artificial vision technologies aimed at achieving more natural and durable visual restoration.

#### PRIMA (Pixium Vision, France)

Pixium Vision, a French company specializing in retinal prosthetics, developed the PRIMA system as an evolution of subretinal implant technology. Building upon foundational research from Stanford University, PRIMA adopts a modular photovoltaic design to enable wireless, light-driven stimulation of retinal neurons.

**Fig. 5:**
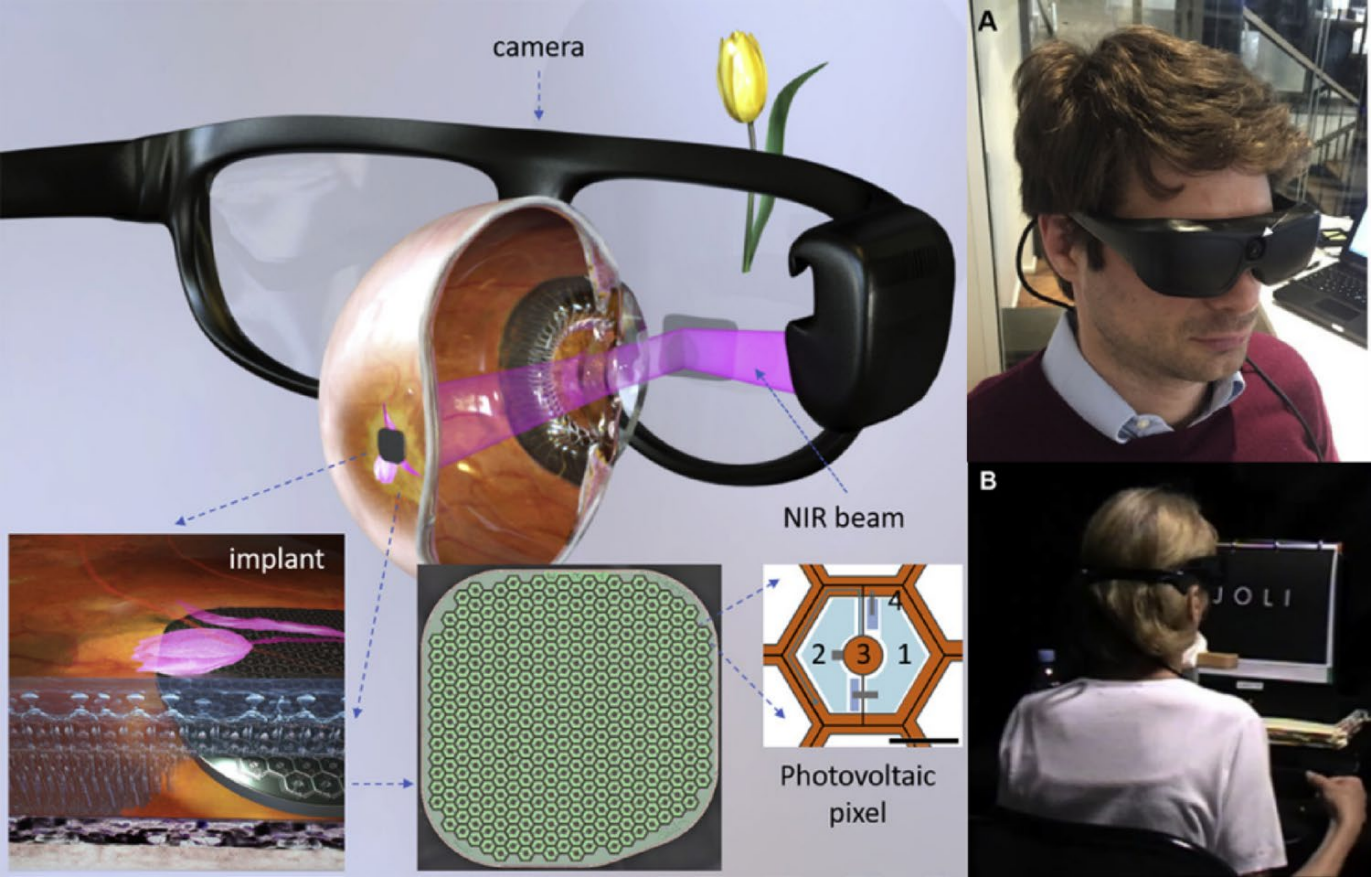
PRIMA subretinal photovoltaic prosthesis system features augmented reality-style video glasses with an integrated camera. The camera captures and processes images, then projects them onto the retina as pulsed near-infrared light. A subretinal wireless photovoltaic array transforms this light into electric current for stimulating retinal neurons. Each pixel in the array contains two diodes connected between active and return electrodes. **A** An image of the video glasses, highlighting the integrated camera. **B** A patient participates in letter recognition and reading tests using the camera. (Reprinted from Palanker et al. [52], under the Creative Commons Attribution License)

As depicted in Fig. 5, PRIMA device consists of a miniaturized implant comprising an array of photovoltaic pixels, each approximately 100 µm in size, fabricated on a biocompatible substrate. Each pixel integrates three photodiodes connected in series to amplify voltage output, enhancing sensitivity to light stimulation. Unlike traditional passive subretinal implants, PRIMA relies on an external augmented reality (AR) glasses system that projects pulsed near-infrared (NIR) light patterns onto the retina. The photovoltaic cells convert the incident NIR light into electrical signals, stimulating the bipolar cells without the need for internal wiring.

By leveraging wireless activation, PRIMA significantly reduces surgical complexity and mechanical stress on ocular tissues. The implant’s modular nature also allows for scalable expansion across larger retinal areas if necessary, accommodating a broader visual field. Clinical trials in 2020 [30, 53, 54], reported a prosthetic visual acuity up to 20/460, slightly outperforming the Alpha IMS, although still below the threshold for functional reading acuity.

One of PRIMA’s key advantages lies in preserving natural eye movement and fixation behavior, as visual perception is directly linked to where the AR glasses project the stimulation. However, reliance on external NIR projection demands precise alignment and synchronization between the implant and external system. Furthermore, as the system depends on external hardware for visual input, the user’s overall experience remains dependent on the performance and ergonomic integration of the AR glasses.

Despite these limitations, PRIMA represents a significant advancement in subretinal prosthesis design, demonstrating the feasibility of wireless, modular stimulation systems. Continued improvements in pixel miniaturization, optical delivery systems, and long-term biocompatibility are anticipated to enhance future iterations of this technology according to prior reports in [52, 55].

#### Boston retinal implant (Harvard/MIT/VA, USA)

The Boston Retinal Implant Project (BRIP) represents a collaborative effort among institutions including Harvard Medical School, MIT, and the Veterans Affairs Boston Healthcare System. The project aims to develop a hermetically sealed, wireless subretinal prosthesis that prioritizes durability, safety, and scalability for long-term human use. The BRIP device released in 2011 [56–58], features a flexible microelectrode array implanted in the subretinal space, interfacing with an externally mounted titanium-encased electronics package. Power and data transmission occur wirelessly via an inductive coupling system, eliminating the need for transcutaneous wires. This approach minimizes infection risks and simplifies surgical procedures compared to earlier wired systems.

The microelectrode array is designed to deliver precise, localized stimulation to surviving inner retinal neurons. Electrodes are coated with iridium oxide (IrO_x_) to enhance charge injection capacity, ensuring safe and efficient stimulation. Early preclinical studies demonstrated stable wireless operation and effective electrical stimulation without significant adverse tissue reactions.

A notable design strength of the BRIP system is its modularity. The implant's architecture allows separate optimization of the internal implant and the external controller, providing flexibility for iterative technological improvements. Although human clinical trials have not yet commenced, extensive preclinical validation has laid the groundwork for future translation.

By emphasizing hermetic encapsulation, biocompatibility, and modular scalability, the Boston Retinal Implant seeks to overcome limitations faced by earlier retinal prostheses, moving closer toward practical, long-term visual restoration solutions.

#### HARP4k retinal prosthesis system (Iridium Medical Technology, Taiwan)

The HARP4k Retinal Prosthesis System, developed by Iridium Medical Technology based in Taiwan in 2023, represents a major leap in subretinal prosthesis resolution and design. HARP4k introduces a flexible implant platform accommodating approximately 4000 microelectrodes, each about 30 µm thick, strategically distributed to match the natural curvature of the retina.

The device operates wirelessly, with power and data transmitted via an external unit that projects near-infrared (NIR) light onto the implant. The flexible design facilitates surgical implantation while minimizing mechanical mismatch with the retinal surface. The high electrode density of the HARP4k enables complex visual tasks such as face recognition and reading larger fonts, surpassing the spatial resolution offered by previous subretinal implants.

Preclinical and early clinical evaluations reported in [59], demonstrated promising functionality, including improved object recognition and navigational abilities. However, adverse effects such as epiretinal gliosis, inflammatory responses, and mild declines in visual acuity following implantation have been observed, necessitating further optimization of the electrode-tissue interface and surgical techniques.

By significantly increasing electrode density and adopting a flexible, retina-conforming design, the HARP4k system pushes the boundaries of subretinal prosthesis capabilities. Ongoing efforts aim to refine biocompatibility, long-term stability, and integration with external image processing systems to achieve more consistent and meaningful visual restoration outcomes.

### Suprachoroidal prosthesis

Suprachoroidal prostheses represent an emerging strategy that places the electrode array within the suprachoroidal space, located between the sclera and the choroid. This anatomical location offers several advantages, including a relatively less invasive surgical procedure, reduced risk of retinal damage, and greater mechanical stability compared to epiretinal and subretinal approaches.

By avoiding direct contact with the delicate neurosensory retina, suprachoroidal implants minimize the risk of retinal tearing or gliosis. Additionally, the larger anatomical space allows for the integration of larger electrode arrays without compromising ocular structures. However, the greater distance between the electrodes and the target retinal neurons results in higher stimulation thresholds and potentially broader current spread, limiting spatial resolution [60–63].

Recent developments in suprachoroidal prostheses focus on optimizing electrode designs to deliver effective stimulation despite the increased separation, as well as improving surgical techniques to ensure consistent electrode positioning and long-term stability. Clinical trials of suprachoroidal devices have demonstrated encouraging outcomes in terms of safety and basic functional vision restoration, supporting their potential as a viable alternative for treating profound vision loss.

#### Gen2 bionic vision (Bionic Vision Technologies, Australia)

Gen2 Bionic Vision represents a significant advancement in the field of suprachoroidal retinal prostheses, building upon the foundational work initiated by Bionic Vision Australia (BVA). Transitioning into Bionic Vision Technologies (BVT), the project evolved from early 24-channel designs to more sophisticated systems emphasizing higher resolution and greater functional outcomes.

As previously reported in [64–66], he Gen2 system features a 44-channel platinum disc suprachoroidal electrode array mounted on a flexible silicone substrate. The intraocular array comprises 44 stimulating platinum electrodes (D: 1.0 mm) and two return electrodes (D: 2.0 mm) on a silicone substrate, with the intraocular component measuring 19 × 8 mm. Interim clinical trial results reported in 2021 demonstrated a favorable surgical safety profile and clinically meaningful improvements in functional vision, including object localization and basic mobility tasks. Complementary real-world assessments in the same cohort further showed improved task performance with the device ON versus OFF, supporting functional benefit beyond laboratory settings. In addition, navigation-oriented processing methods have been explored on the Gen2 platform, reporting enhanced navigational outcomes with depth-based vision processing. Taken together, these results indicate that the Gen2 suprachoroidal approach advances the safety and functional utility of retinal prostheses while preserving a comparatively less invasive surgical pathway established by the BVA program.

#### Phoenix99 (University of New South Wales, Australia)

The Phoenix99 retinal prosthesis, developed by researchers at the University of New South Wales and affiliated institutions in 2014, represents an advanced suprachoroidal visual prosthesis designed for enhanced safety, stability, and long-term function.

**Fig. 6:**
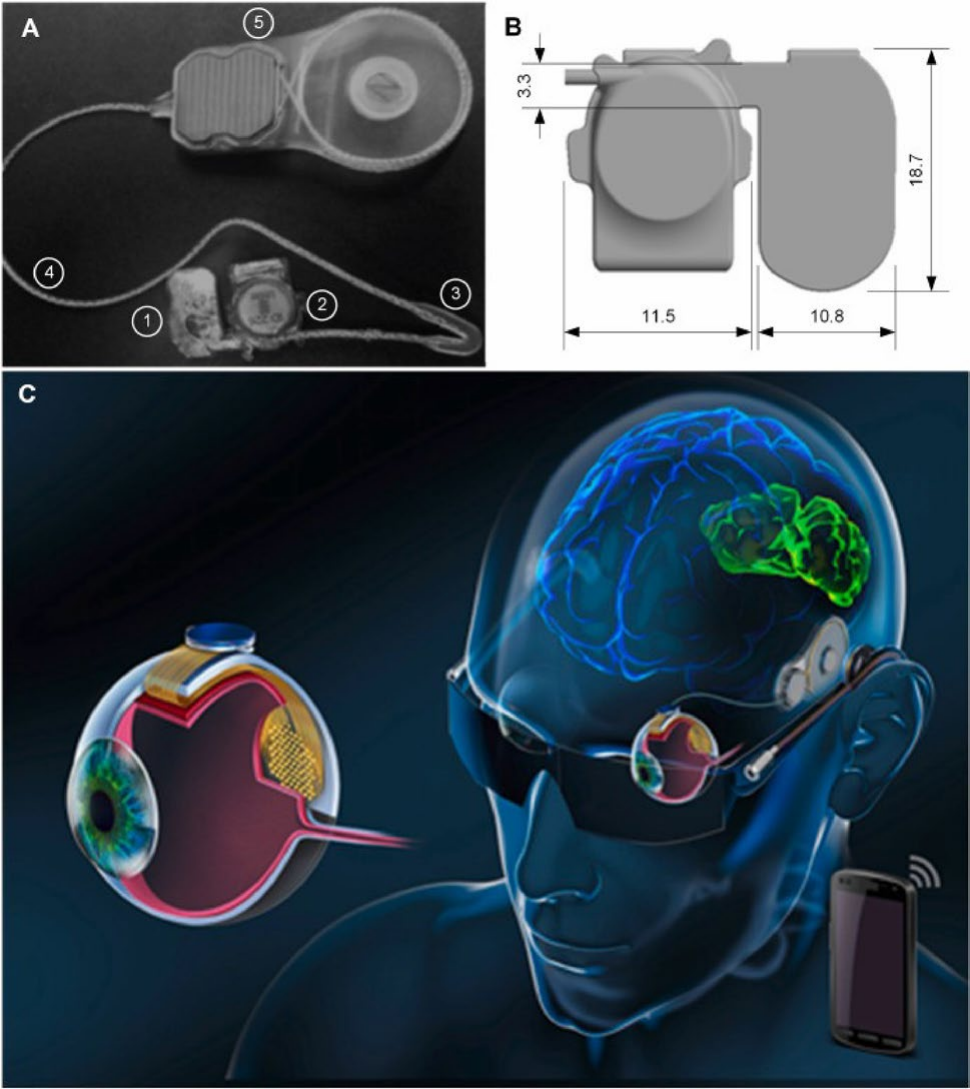
The Phoenix99 suprachoroidal retinal prosthesis system, showing **A** Showcases the complete implant featuring the electrode array, VS capsule, silicone grommet, flexible cable, and TI. **B** Displays a 3D rendering of the VS capsule, highlighting its critical dimensions. **C** Presents an artistic representation of the system's placement within the eye (Reprinted from Eggenberger et al. [69], under the Creative Commons Attribution License)

According to [67–69], the Phoenix-99 architecture comprises a total of 99 electrodes configured as 98 stimulating electrodes plus a common return, integrating a flexible array that conforms to the curvature of the eye and a subdermal receiver/telemetry capsule for wireless power and data (Fig. 6).

**Fig. 7:**
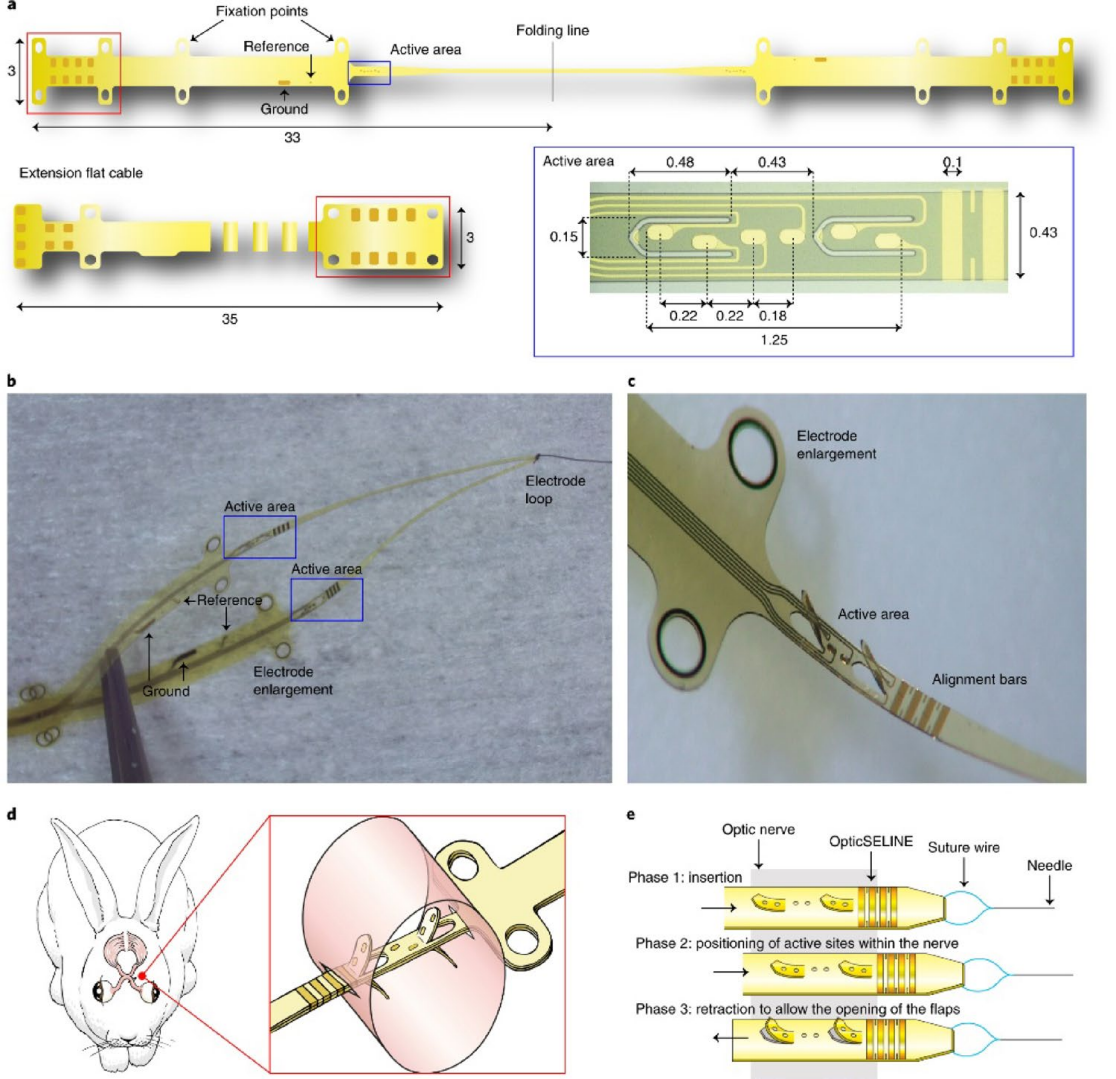
The OpticSELINE intraneural optic-nerve implant, showing **a** device layout with fixation points, reference/ground contacts, active area, folding line, and extension flat cable (inset: active-area design); **b** and **c** photographs of the implant with alignment bars and electrode loop; **d** schematic of placement at the optic disc in the rabbit; and **e** the insertion–deployment sequence using a suture wire to open the flaps and position active sites within the nerve (Reproduced with permission from Gaillet et al. [70], Nature Biomedical Engineering, Springer Nature)

Long-term preclinical implantation in sheep demonstrated stable device positioning, low inflammatory response, and consistent stimulation thresholds over chronic periods, with imaging and histopathology supporting biocompatibility and mechanical stability of the suprachoroidal approach. The system’s modular, fully implantable design provides a pathway for future upgrades in channel count and on-device processing while maintaining a minimally invasive surgical pathway (Fig. 7).

#### STS NIDEK (Nidek Co., Japan)

The Suprachoroidal-Transretinal Stimulation (STS) system, developed by Nidek Co., Ltd. in collaboration with academic partners in Japan, represents an alternative suprachoroidal prosthesis approach. The STS strategy targets the retina by delivering electrical stimulation across the choroid and retinal layers from a suprachoroidal electrode array.

The STS system comprises a flexible electrode array positioned within the suprachoroidal space, coupled with an implantable receiver-stimulator unit. The electrode array consists of 49 platinum electrodes, each approximately 500 µm in diameter, arranged over an area measuring approximately 5 mm × 5 mm. Power and stimulation commands are transmitted wirelessly to the implant using transcutaneous radiofrequency (RF) communication. The electrode configuration is designed to stimulate inner retinal neurons through controlled current spread across the choroidal vasculature, providing a broader and deeper stimulation field.

Early clinical trials demonstrated in 2015 [71, 72] that the STS system enabled patients with profound vision loss to perceive light, localize objects, and improve mobility. Compared to conventional epiretinal or subretinal devices, the STS approach offers advantages in terms of surgical accessibility, reduced mechanical stress on the retina, and improved device stability. However, spatial resolution remains limited due to the indirect stimulation pathway.

The STS Nidek project highlighted the feasibility of using suprachoroidal-transretinal electrical stimulation to evoke functional visual responses, broadening the range of strategies available for artificial vision restoration. Ongoing research focuses on optimizing electrode designs and stimulation parameters to enhance spatial specificity and visual acuity.

### Optic nerve stimulation

Optic nerve stimulation represents an emerging strategy for vision restoration that bypasses degenerated retinal structures and directly targets the optic nerve to elicit visual percepts. By stimulating the optic nerve fibers, this approach aims to restore vision in patients with severe retinal degeneration where photoreceptors and inner retinal layers are largely nonfunctional.

Various methods for optic nerve stimulation have been explored, including epineural, intraneural, and cuff electrode configurations. Challenges include the complex anatomical organization of optic nerve fibers, which makes achieving high spatial resolution difficult, and the risk of eliciting non-visual sensations or causing nerve damage. Nonetheless, recent advancements in electrode design, signal processing, and surgical techniques have revitalized interest in this strategy as a viable alternative for restoring some degree of functional vision.

#### Extraneural (cuff) electrode

Extraneural systems wrap a self-sizing spiral cuff around the optic nerve to excite bundles without penetrating the parenchyma. In the landmark human study by Veraart and colleagues, a blind volunteer with retinitis pigmentosa was chronically implanted with a spiral cuff; electrical stimulation reliably produced localized phosphenes whose position and appearance depended on stimulation parameters (amplitude, pulse width, contact selection). With systematic parameter changes, the team could elicit topographically distributed percepts and even simple geometric patterns, establishing a functional mapping between cuff contacts and perceived location in the visual field as shown in [73].

Subsequent work in refined the surgical approach from an intracranial placement to an intraorbital, medial transconjunctival route, reducing invasiveness while preserving access to the nerve; this transition enabled multi-contact cuffs (4- and 8-contact designs) and expanded opportunities for current steering to shape percepts. Training studies in [74], further showed that an implanted volunteer could localize, discriminate, and grasp objects using optic-nerve stimulation, underscoring translational potential for orientation and reach-to-grasp behaviors in controlled tasks.

From a device standpoint demonstrated in [75], spiral cuffs are prized for long-term mechanical stability and nerve viability, and they serve as a platform for multi-contact, current-steered stimulation that trades fine selectivity for lower surgical risk compared with intraneural approaches. Reported human data describe broad visual-field coverage with contact-dependent phosphene size/brightness and frequent white/yellow coloration, consistent with coarse recruitment of fiber populations near the nerve surface.

#### Intraneural/penetrating electrodes

Intraneural strategies insert microelectrodes into the intraorbital or intracranial optic nerve to target fascicles more selectively. In animal models, penetrating arrays evoke robust electrically-evoked cortical potentials (EEPs) whose latency and amplitude depend on pulse parameters and stimulation mode (monopolar vs. bipolar), demonstrating controllable recruitment dynamics [76]. Beyond evoking responses, retinotopic (visuotopic) organization can be addressed: in cats, a penetrating array just posterior to the globe mapped to corresponding regions of visual cortex, with visual-field shifts observed as electrode depth progressed across the nerve: evidence that intraneural placement affords spatial addressing within the nerve cross-section [77].

More recently, intraneural stimulation in rabbits with an intracranially placed array produced spatially selective activation patterns in primary visual cortex recorded by electrocorticography (ECoG), supported by numerical modeling of current spread and fiber recruitment; as reported in [70] this line of work argues that intraneural arrays can, in principle, scale spatial selectivity beyond what’s typical for extraneural cuffs. In terms of safety/operating windows, reported charge-density thresholds [78] are on the order of tens of µC cm^−2^ for short, symmetric biphasic pulses, compatible with known safety limits for chronic neural interfaces when carefully managed, though long-term axonal health, micromotion, and glial remodeling remain central considerations.

#### Optic-disc implantation: AV DONE (Nidek Co., Japan)

The AV-DONE approach targets the optic disc by inserting fine wire electrodes directly into the nerve head to elicit visual percepts in patients with advanced outer retinal degeneration. In the reported human case, the implant comprised three platinum wire electrodes (≈ 50 µm diameter) inserted into the optic disc; stimulation at individual contacts produced localized phosphenes with circular, elliptical, or linear shapes, and no major postoperative complications were noted during follow-up. Representative thresholds (0.25 ms/phase, 320 Hz) were ~ 30, 5, and 70 µA for the three contacts, respectively.

A subsequent device iteration described a multi-wire configuration with seven 50 µm stimulating wires and a common return integrated on a ~ 2.0 mm silicone board and deployed via a ~ 100 µm insertion rod, enabling a single vitreous entry and stable fixation at the disc. Preclinical implantation demonstrated consistent visually evoked responses and supported the feasibility of increasing channel count while maintaining surgical simplicity as depicted in [79, 80].

Although the resolution achieved remains limited compared to natural vision, the AV-DONE project provided critical proof-of-concept evidence supporting direct optic nerve stimulation as a viable strategy for visual restoration. Ongoing developments focus on improving the spatial selectivity of stimulation, optimizing electrode arrangements, and expanding the range of functional visual capabilities.

### Visual cortex stimulation

Cortical prostheses target primary visual cortex (V1) to evoke retinotopically organized phosphenes when retinal or optic-nerve options are not feasible. Two main strategies are used: epidural/subdural surface arrays (surgical simplicity, broad coverage) and intracortical microelectrode arrays (higher spatial selectivity).

#### ORION (Second Sight, USA)

The ORION device, created by Second Sight, is a significant advancement in the field of visual prosthetics. This device includes a built-in camera that captures external visual information, and an image-processing unit that transforms these images into electrical signals. These signals are then transmitted to an electrode array implanted between the skull and brain, allowing the neural system to receive and process visual information. The ORION system is noteworthy for its ability to address a broader range of diseases, strategic placement of the implant for optimal neural interaction, and enhanced capacity to enable patients to navigate their surroundings. The precise dimensions of the implant were not specified in the available documentation. The ORION system consists of an array of 60 electrodes.

In 2020 study [81–83], early human studies report reliable phosphene perception across multiple contacts and improvements in basic orientation and localization tasks with the system ON, alongside iterative refinements in encoding and user training to improve percept stability and usefulness in controlled settings. Performance varies across users, reflecting differences in electrode coverage, cortical anatomy, and adaptation time; ongoing work targets spatiotemporal encoding and scene simplification to increase effective resolution.

#### ICVP (Illinois Institute of Technology, USA)

The Illinois Institute of Technology's Intracortical Visual Prosthesis (ICVP) project marks a noteworthy achievement in developing advanced visual prosthetics applied in 2023 according to [84]. This project employed wireless floating microelectrode arrays, with each module comprising six electrodes measuring 2 mm × 2 mm. The wireless architecture of the system eliminates the need for wires or connectors to traverse the scalp, thereby reducing patient discomfort and infection risk. Additionally, the ICVP device enables direct transmission of camera-captured images to the brain, bypassing conventional visual pathways. This innovation holds great potential for providing transformative solutions for individuals with severe visual impairments.

Developing and implementing advanced neural interfaces necessitate a well-balanced emphasis on both efficacy and safety. It is essential to ensure that these innovative technologies can be safely integrated into clinical applications, thereby significantly enhancing the lives of individuals with visual impairments.

## Recent trends in artificial retina technology

### Flexible materials

**Fig. 8:**
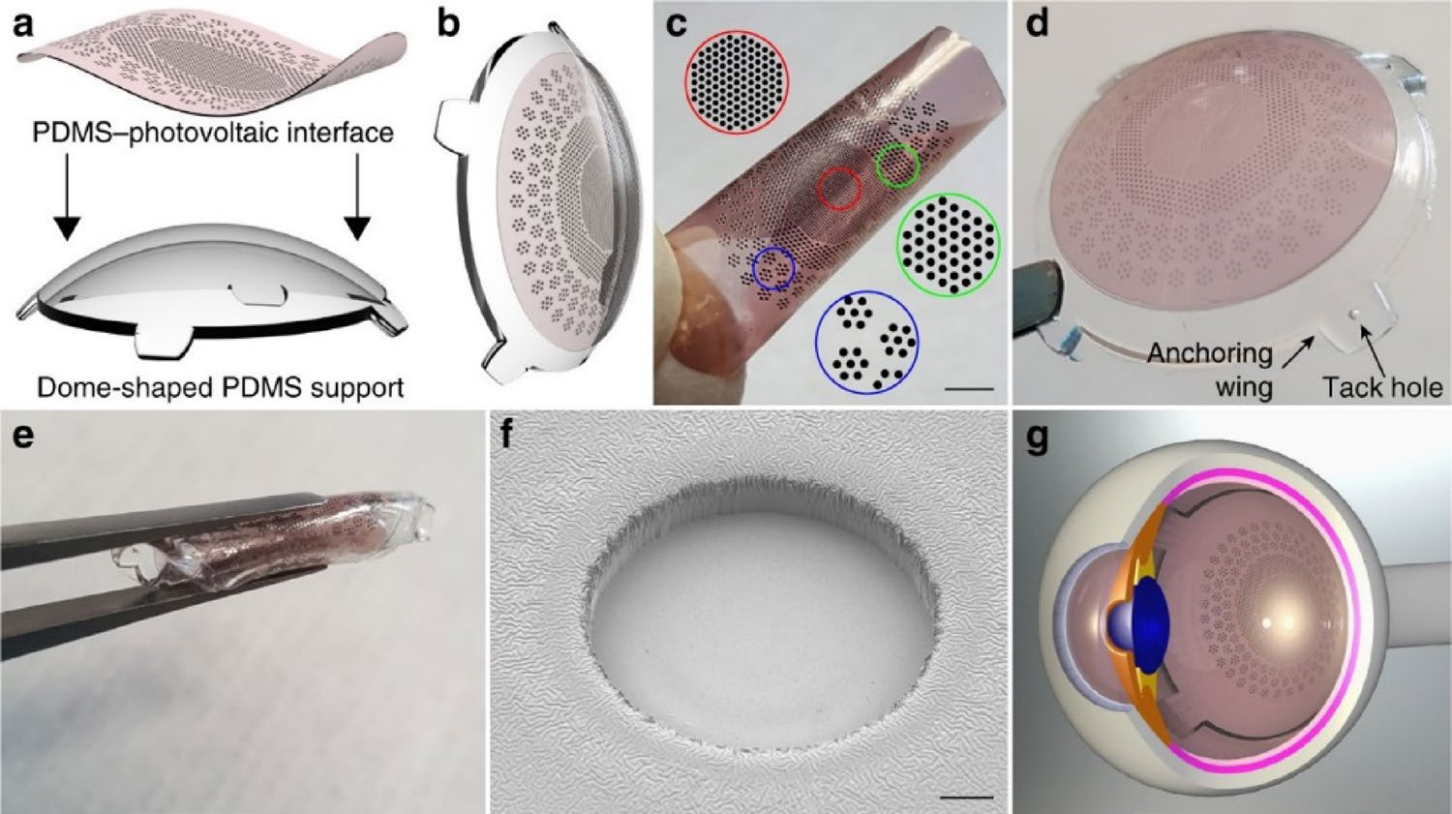
The foldable photovoltaic wide-field epiretinal prosthesis, showing **a** A 3D model of the PDMS-interface and its dome-shaped support. **b** The prosthesis post-bonding of the PDMS-interface to the support. **c** The PDMS-photovoltaic interface with variably sized and dense electrode areas: central (5 mm diameter, 967 electrodes), first ring (8 mm, 559 electrodes), and second ring (12.7 mm, 719 electrodes). **d** Image of POLYRETINA featuring four anchoring wings for attachment. **e** POLYRETINA folded pre-injection. **f** Scanning electron microscope view of a photovoltaic pixel. **g** 3D model of the prosthesis in epiretinal position (Reprinted from Ferlauto et al. [86], under the Creative Commons Attribution License)

The use of flexible materials in artificial retinal devices is increasingly being recognized as advantageous, particularly given the spherical nature of the human eye. These materials enable devices to maintain a uniform contact with the inner wall of the retina, which is essential for optimal performance. Traditionally, polyimide has been the primary material used in constructing and packaging artificial retinas. However, recent technological advancements have led to the development of flexible image sensors for artificial retinas using materials such as MoS2 layered on polyimides as reported in [85]. For instance, as shown in Fig. 8, Ferlauto et al. [86] developed a notable breakthrough from EPFL in Switzerland of a fully foldable artificial retinal electrode that incorporates P3HT, known for its photoelectric effects, and polydimethylsiloxane (PDMS), a flexible polymer. This combination allows for extensive coverage of the retinal area while maintaining the ease of insertion of the device owing to its foldable structure.

**Fig. 9:**
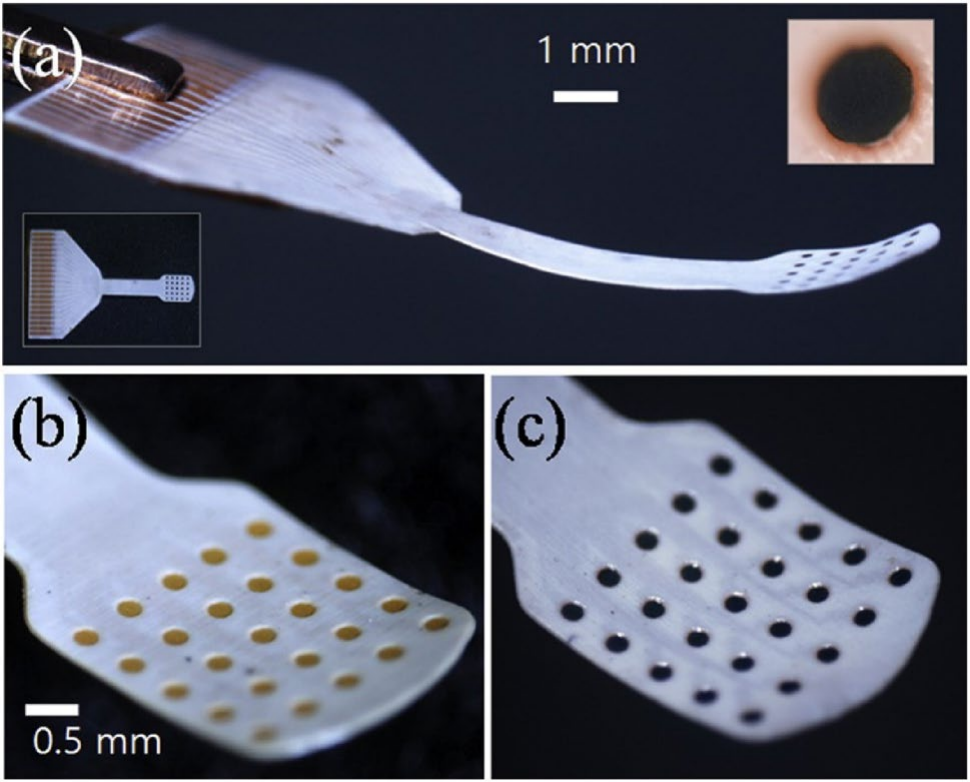
**a** A 25-channel neural electrode array made from LCP, designed with a thermoformed curvature to fit the target organ or tissue (inset shows a top view; scale bar: 5 mm). The electrode surface is available in **b** electroplated gold or **c** Iridium Oxide (Reprinted from Jeong et al. [87], with permission from Elsevier)

Research in the field of bio-implantable devices has evolved to incorporate a wider range of flexible materials in addition to polyimide and PDMS. This expansion seeks to enhance the long-term durability of these devices by integrating simple circuits and sensors into their materials. However, this requires advancements in the packaging technology. Current studies have explored the use of biocompatible and flexible materials such as Liquid Crystal Polymer (LCP) films [87] (Fig. 9), cyclic olefin copolymers (COC) [88], and perfluoroalkoxyalkanes (PFA) [89] for packaging purposes. The use of these materials is expected to significantly improve the longevity and functionality of bioimplantable devices, representing a significant breakthrough in artificial retinal technology.

### Photovoltaic structure

In the domain of artificial vision, the intricate process of configuring and operating individual electrodes in arrays poses considerable challenges, particularly in attaining extensive high-resolution capabilities. One noteworthy strategy for addressing this issue is the Alpha IMS of the Retinal Implant, which integrates circuits with active electrodes to mitigate complex wiring problems. However, a significant drawback emerges with the CMOS chip’s post-implantation failure, as reported by [90], highlighting a critical durability concern. In contrast, Second Sight's Argus II, which employs passive electrodes without a CMOS chip, exhibited enhanced durability compared to Alpha-IMS, indicating that active electrode technology necessitates a robust solution for integrated circuit packaging.

**Fig. 10:**
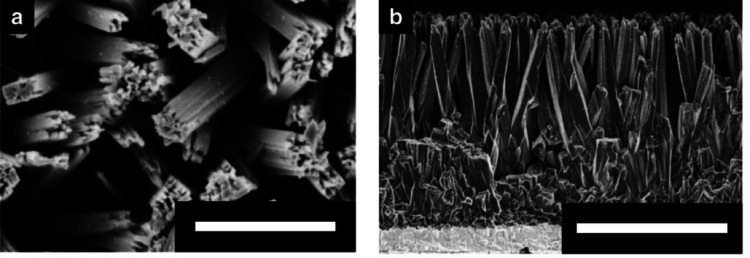
Photovoltaic capabilities of Au–TiO_2_ NW arrays are demonstrated through **a** top-view and **b** side-view scanning electron microscope images (Reprinted from Tang et al. [92], under the Creative Commons Attribution License)

Recent advancements in the field have focused on the utilization of materials with photovoltaic effects to eliminate the need for complex wiring and simplify device construction. In previous research [91], significant breakthrough in this area was achieved by CNRS in France, which successfully stimulated retinal nerve cells using TiO_2_ nanotubes that generated current upon exposure to light. To enhance this approach, Fudan University in China improved the photoconversion efficiency by incorporating gold (Au) nanoparticles into the TiO_2_ nanowires according to [92] as shown in Fig. 10. Additionally, the Ecole Polytechnique Federale de Lausanne (EPFL) as shown in [86] developed a state-of-the-art artificial retinal device utilizing the photovoltaic effect of P3HT (poly(3-hexylthiophene-2,5-diyl)), with remarkable results. The application of these materials is particularly advantageous, as they are conducive to the creation of flexible substrate-based artificial retinal devices, opening up new avenues for future innovations in the field.

**Fig. 11:**
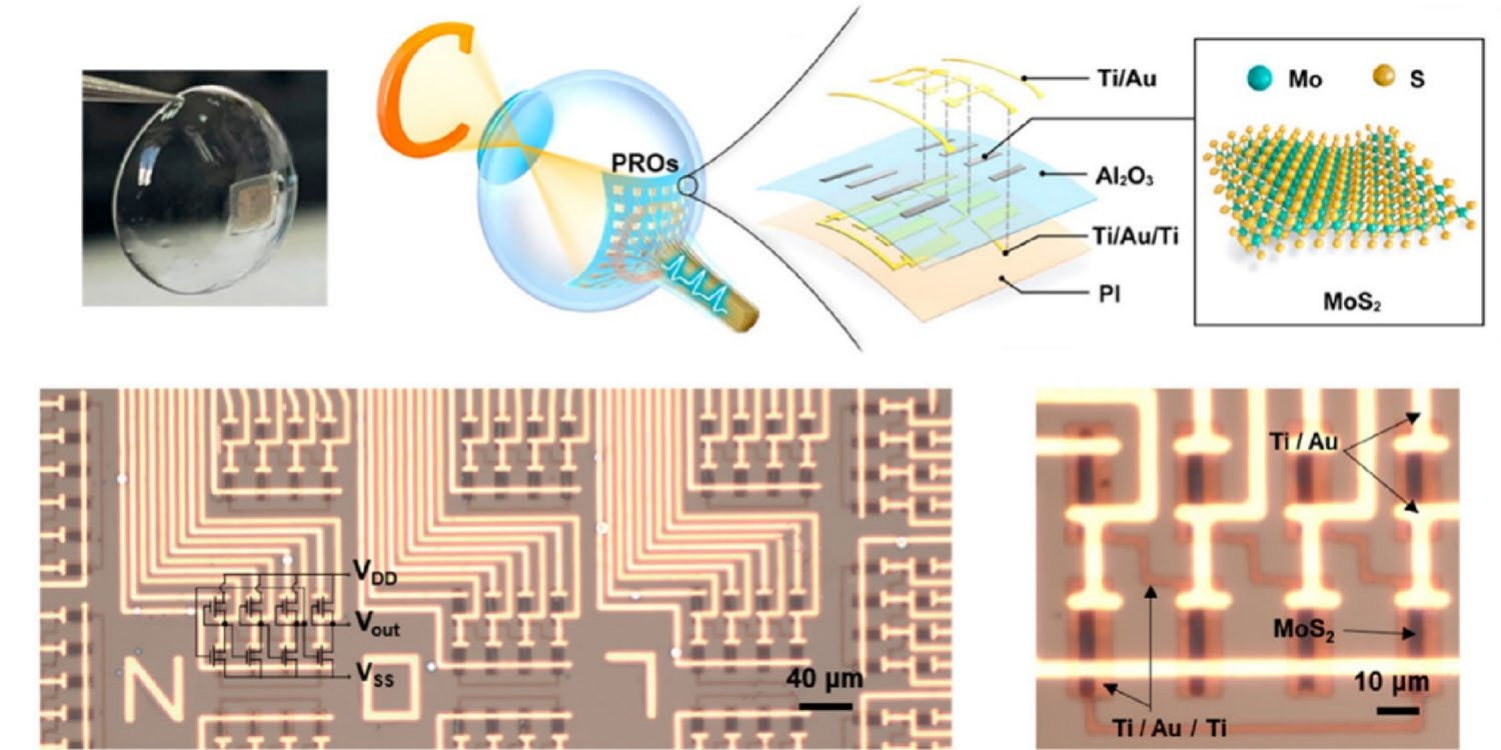
The MoS_2_-based artificial retina, showing Top-left: Features a photo of the flexible, implantable MoS_2_ retina on a contact lens. Top-right: Presents a schematic of the PRO array. Top-middle: Details the layered composition of a single PRO and the atomic structure of the MoS_2_ channel. Bottom: Displays optical micrographs of the PRO array and an individual PRO, with a schematic of the PRO’s device structure, including supply voltages (VDD, VSS) and the output voltage (Vout) (Reprinted from Li et al. [93], with permission from the American Chemical Society)

As reported by [93], the development of flexible photoresponsive ring oscillators (PROs) using MoS_2_ for artificial retinas has contributed significantly to the evolving landscape (Fig. 11). PROs effectively convert natural light into electrical pulses that are compatible with the human visual system and operate with ultralow power consumption, approximately 500 times lower than that of current silicon-based devices, and function at supply voltages below 1 V. The simplicity, high efficiency, and adaptability of these PROs make them promising candidates for future clinical trials of vision restoration, representing a significant advancement in the pursuit of effective artificial vision solutions.

### AR glasses

In 2019, Cellico, a South Korean company, emerged as a pioneer in the field of solutions for visual impairment by developing electronic eyes or artificial retinas. The company's primary focus is on a conscious artificial retinal medical device that aims to restore vision by implanting high-resolution stimulation devices into the damaged photoreceptor layer of the eye. According to prior reports [94, 95], Cellico inserts an image sensor chip into the damaged photoreceptor layer, which is designed to detect light and convert it into electrical signals transmitted to the brain via the optic nerve. In addition, the company has also created AR glasses specifically designed for patients in the early to mid-stages of retinal disease. These glasses have a camera that captures a broad field of view, processes images in real-time, and displays them in a format that is perceivable by the patient's retina. Furthermore, AR glasses incorporate a magnetic induction coil for wireless charging and offer customization options that align with user style preferences. The integration of AR glasses with artificial retinal devices offers several advantages. First, these glasses capture a wider field of view, significantly enhancing the user's visual experience compared with traditional artificial retinas. They process images in real-time, providing users with immediate and updated visual information that is essential for interacting with dynamic surroundings.

In addition, the ability to adjust the display format to suit individual retinal impairments enhances the effectiveness of the device.

### 3D structured electrodes

To create high-resolution artificial retinal devices such as Pixium Vision's PRIMA device, it is essential to guarantee that the electric current applied to the electrodes is concentrated and targets a narrowly defined area of retinal nerve cells. However, a significant challenge arises when attempting to stimulate a small area on the retina surface without enlarging the stimulation size, as this may limit the ability to reach and stimulate cells deeper within the retina. To overcome this obstacle, researchers at Stanford University, the creators of the PRIMA device [96], developed an innovative honeycomb-shaped 3D return electrode. This novel design focuses on electrical stimulation in a confined area while extending its reach deeper into retinal layers.

**Fig. 12:**
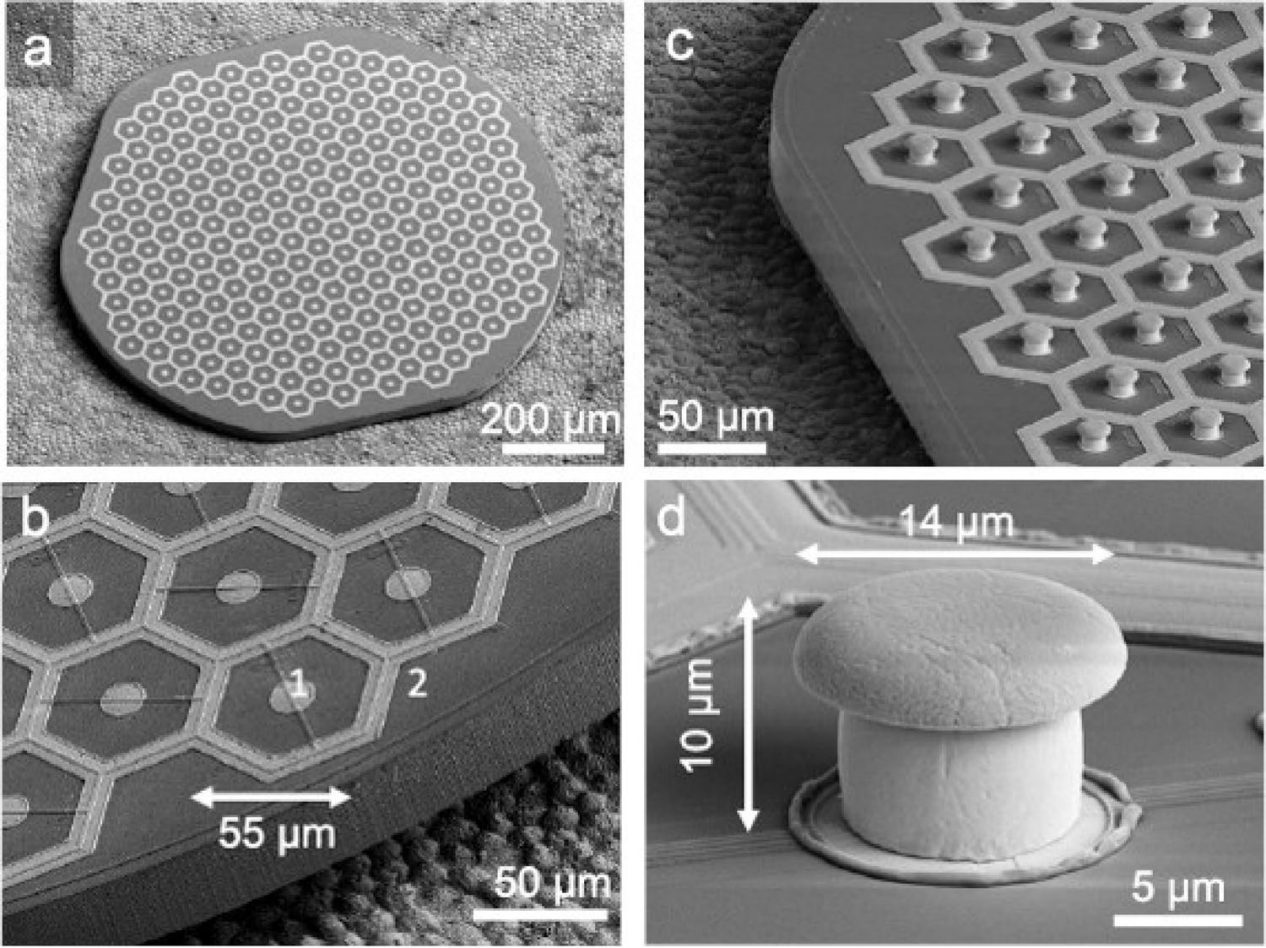
Photovoltaic microelectrode arrays with hexagonal pixel arrangement, showing **a** A 1 mm wide implant containing 250 pixels; **b** A close-up showing the central active electrode (14 µm diameter) and smaller return electrodes (9 µm); **c** A similar array with pillar electrodes; **d** A detailed view of a single pillar electrode, 10 µm tall with a 14 µm wide SIROF-coated cap (Reprinted from Ho et al. [97], under the Creative Commons Attribution License)

As shown in Fig. 12, Ho et al. [97] systematically compared flat versus pillar subretinal photovoltaic pixels and showed that elevating the active electrode as a pillar with a surrounding return ring tightened current spread and sharpened the elicited percepts relative to flat pixels.

**Fig. 13:**
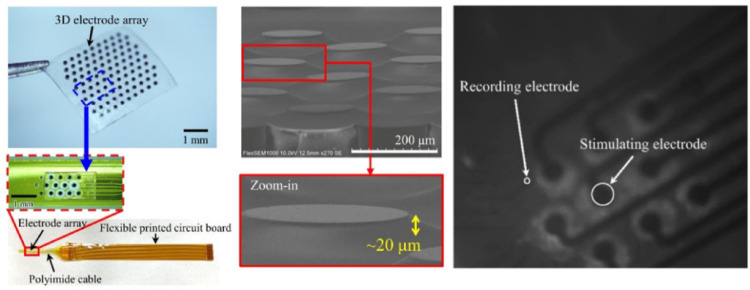
A 3D microelectrode array was created for subretinal stimulation. Part of this array was modified with a custom polyimide cable and a flexible printed circuit board for in-vitro tests. Scanning electron microscope images showcase the 3D structure of the electrodes. In in-vitro experiments, the position of the subretinal electrodes relative to the 59 recording electrodes is detailed, highlighting the varying distances (175, 350, 525, 700 µm) between the stimulating and recording electrodes, indicated by white (recording) and red (stimulating) dots (Reprinted from Seo et al. [98], with permission from Springer Nature)

Seo et al. [98], conducted a study in 2023 that involved the development of a 3D microelectrode array measuring 20 µm in height. A microelectrode array was fabricated using IrO_x_ for subretinal stimulation (Fig. 13). To facilitate in vitro testing, researchers attached a custom polyimide cable and a flexible printed circuit board to the array. In vitro experiments performed by Seo et al. demonstrated the strategic placement of subretinal electrodes in relation to 59 recording electrodes. The configuration revealed varying distances between the stimulating and recording electrodes, emphasizing the need for precision when targeting specific regions of the retina during nerve cell stimulation to prevent expansion of the stimulated area.

**Fig. 14:**
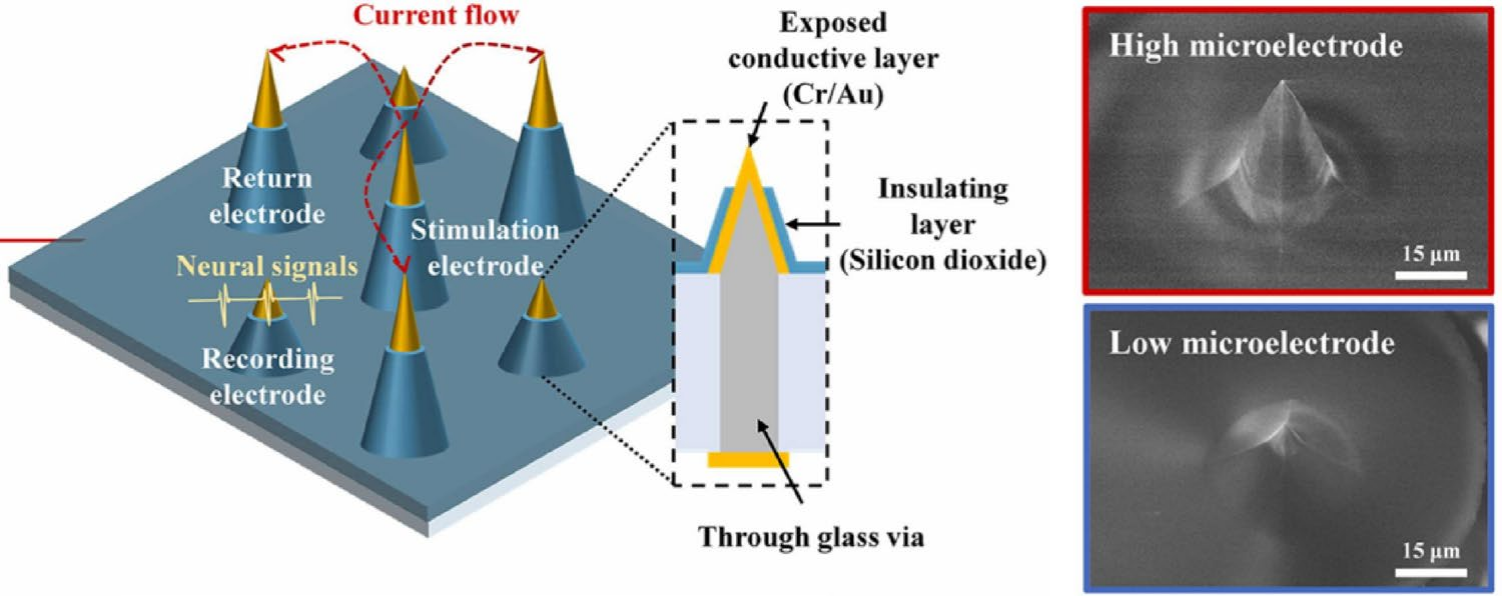
The proposed microelectrode array (MEA) design features a unit, highlighted in red, comprising one stimulation electrode, three return electrodes for electric confinement, and three shorter recording electrodes. The electrodes have silicon dioxide-insulated sidewalls with conductive tips, connected through glass via to a base metal layer Reprinted from Shin et al. [99], with permission from Elsevier)

Shin et al. [99], exhibited authority over the elevation of microelectrodes through silicon deep reactive ion etching, enabling electrical connections through glass vias (TGVs) for individual electrode addressing (Fig. 14). Aligning with the development of high-resolution artificial retinal devices such as Pixium Vision’s PRIMA, their intent was to confine electrical stimulation within a specifically defined retinal region. This objective was accomplished by deploying adjacent microelectrodes as regional returns around a stimulation electrode, mirroring the challenge of stimulating retinal surface areas without enlarging the stimulation size, which is critical for reaching deeper retinal cells.

Improvements in three-dimensional electrode designs and investigations of novel materials are crucial for the advancement of artificial retina technology. The development of electrodes capable of delivering more concentrated and precise electrical stimulation is vital for improving the overall resolution and efficiency of artificial retinal devices. This emphasis on enhancing the accuracy and intensity of stimulation demonstrates the ongoing dedication within the field to increasing the functionality of these devices and their potential to benefit individuals with visual impairments.

## Discussion

Artificial vision prostheses have progressed from proof-of-concept systems to platforms that deliver reproducible phosphenes and task-level gains in selected indications, yet their long-term clinical value hinges on durability, effective resolution, surgical practicality, and user training.

Clinical efficacy across targets. Epiretinal systems such as Argus II [20] demonstrated real-world improvements in light perception, motion detection, and object localization over multi-year follow-up, though with limited acuity. Subretinal photovoltaics (PRIMA) [54] restored central letter recognition and achieved prosthetic acuity in the 20/460 range, with durability testing and multi-year clinical updates supporting continued function. Suprachoroidal devices advanced rapidly: the second-generation 44-channel system [64, 65] reported favorable safety and functional gains in both clinical metrics and real-world tasks, while STS [72] approaches broadened the candidate pool with less invasive access. Beyond the eye, optic-nerve cuffs and penetrating electrodes [70, 73–77, 100] produced localized phosphenes and cortical responses in humans or animals, and cortical programs (ORION, ICVP) [81, 84] showed ON-vs-OFF benefits and patterned percepts in early studies. Collectively, these results confirm feasibility but also delineate the ceiling imposed by biology, interfaces, and encoding.

Simply increasing channel count does not linearly translate to functional acuity because of current spread, retinal circuitry, and cortical integration. Modeling and human or animal work around pixel size and pitch [55] highlight a pixel-size floor below which effective gains diminish. Engineering strategies to tighten activation volumes, including pillar electrodes and regional returns [96–98], improve confinement and cell-scale addressing in preclinical settings. These device-level gains must be coupled to spatiotemporal encoding and task-specific training to convert hardware resolution into usable vision.

Device survival in a saline, micromotional environment remains a defining constraint. Post-implant CMOS failures in active subretinal chips underscore the need for robust hermetic packaging and interconnect reliability [90]. Parallel progress in encapsulation and substrates, LCP, COC, and PFA films [87–89], targets moisture barrier performance, mechanical conformity, and stable electrode interfaces for decade-scale operation. Hermetic design lessons from the Boston Retinal Implant [56–58] remain instructive as systems scale.

Selectivity and safety trade-offs by site. Epiretinal arrays risk variable electrode–retina distance and heat with higher currents [23]; subretinal arrays lower thresholds but require delicate retinal detachment and reattachment [60–63]; suprachoroidal arrays simplify access and stability yet face larger electrode–neuron gaps and broader current spread [64–66]. Optic-nerve and cortical routes bypass diseased retina entirely, trading higher selectivity potential (penetrating/cortical microelectrodes) for greater invasiveness and device complexity [70, 73–77, 81–84, 100]. Across platforms, reported adverse events largely track procedure-, device-, and stimulation-related categories; consolidating safety reporting enables fair cross-comparison [28, 72, 81].

Wireless power/data and external processing are now standard for clinical viability. Photovoltaic stimulation offloads intraocular wiring and simplifies implants (PRIMA; wide-field epiretinal PV) [52, 86], while AR-style optics and head-worn processing pipelines can expand the functional field and enable adaptive encoding for low vision [52, 94, 95]. These architectures emphasize system-level co-design: optics, encoding, and implant physics must be tuned together to maximize information throughput to the brain.

Emerging 3D electrodes with regional returns and recording-capable geometries point to closed-loop possibilities: monitoring responses while stimulating to stabilize percepts and reduce variability [97–99]. At the cortical level, intracortical arrays promise higher spatial addressing, but long-term stability and safe charge delivery will determine translational success [84].

The next phase should prioritize: (1) packaging and interconnect reliability validated to 10-year targets; (2) constrained-spread electrodes and cell-type-aware encoding to convert hardware pixels into perceptual features; (3) standardized outcomes that include real-world navigation and scene parsing in addition to lab tasks; and (4) human-factors integration of AR optics, power, and training workflows. Converging advances across materials, device physics, and neural encoding will be required to achieve stable, functionally meaningful artificial vision beyond today’s capabilities.
